# Ultra-Fast Analysis of Plasma and Intracellular Levels of HIV Protease Inhibitors in Children: A Clinical Application of MALDI Mass Spectrometry

**DOI:** 10.1371/journal.pone.0011409

**Published:** 2010-07-01

**Authors:** Jeroen J. A. van Kampen, Mariska L. Reedijk, Peter C. Burgers, Lennard J. M. Dekker, Nico G. Hartwig, Ineke E. van der Ende, Ronald de Groot, Albert D. M. E. Osterhaus, David M. Burger, Theo M. Luider, Rob A. Gruters

**Affiliations:** 1 Department of Neurology, Erasmus MC, Rotterdam, The Netherlands; 2 Department of Paediatrics, Erasmus MC – Sophia, Rotterdam, The Netherlands; 3 Department of Virology, Erasmus MC, Rotterdam, The Netherlands; 4 Department of Internal Medicine, Erasmus MC, Rotterdam, The Netherlands; 5 Department of Paediatrics, University Medical Centre Nijmegen, Nijmegen, The Netherlands; 6 Department of Clinical Pharmacy, University Medical Centre Nijmegen, Nijmegen, The Netherlands; University of California, San Francisco, United States of America

## Abstract

HIV protease inhibitors must penetrate into cells to exert their action. Differences in the intracellular pharmacokinetics of these drugs may explain why some patients fail on therapy or suffer from drug toxicity. Yet, there is no information available on the intracellular levels of HIV protease inhibitors in HIV infected children, which is in part due to the large amount of sample that is normally required to measure the intracellular concentrations of these drugs. Therefore, we developed an ultra-fast and sensitive assay to measure the intracellular concentrations of HIV protease inhibitors in small amounts of peripheral blood mononuclear cells (PBMCs), and determined the intracellular concentrations of lopinavir and ritonavir in HIV infected children. An assay based on matrix-assisted laser desorption/ionization (MALDI) - triple quadrupole mass spectrometry was developed to determine the concentrations of HIV protease inhibitors in 10 µL plasma and 1×10^6^ PBMCs. Precisions and accuracies were within the values set by the FDA for bioanalytical method validation. Lopinavir and ritonavir did not accumulate in PBMCs of HIV infected children. In addition, the intracellular concentrations of lopinavir and ritonavir correlated poorly to the plasma concentrations of these drugs. MALDI-triple quadrupole mass spectrometry is a new tool for ultra-fast and sensitive determination of drug concentrations which can be used, for example, to assess the intracellular pharmacokinetics of HIV protease inhibitors in HIV infected children.

## Introduction

HIV protease inhibitors exert their action inside cells that are susceptible to HIV infection. The intracellular pharmacokinetics of these drugs, its relation to the plasma pharmacokinetics, and its relation to drug efficacy and toxicity is therefore an active area of research [1]–[10]. However, determination of the intracellular levels of antiretroviral drugs is an analytical challenge, because of the small amount of target cells, e.g. peripheral blood mononuclear cells (PBMCs), which can be obtained from a patient's blood sample. Approximately one million PBMCs can be obtained from 1–2 mL blood. This amount of PBMCs has a total intracellular volume of ∼0.4 µL, while 500 – 1000 µL plasma can be obtained from that same blood sample. As a consequence, much is known about the plasma pharmacokinetics of HIV protease inhibitors in HIV infected children, but no information is available on the intracellular levels of HIV protease inhibitors in this group of patients [11]–[14].

Here, we describe a new assay for fast and sensitive determination of HIV protease inhibitor levels in one million PBMCs and 10 µL plasma, and its application in HIV infected children treated with Kaletra, a co-formulation of the HIV protease inhibitors lopinavir and ritonavir. To this end we used the novel matrix-assisted laser desorption/ionization (MALDI) – triple quadrupole mass spectrometry technique [15]–[23].

## Materials and Methods

### Ethics Statement

This research has been approved by the Medisch Ethische Toetsings Commissie of the Erasmus MC (#206.382/2001/238). Written informed consent was obtained from the patients' parents or legal guardians.

### Chemicals and solvents

Methotrexate (Emthexate PF for parenteral administration, PharmaChemie BV, Haarlem, the Netherlands), saquinavir (donated by F.Hoffmann La-Roche, Basel, Switzerland), indinavir (donated by Merck, Rahway, NJ, USA), nelfinavir (donated by Pfizer, Groton, CT, USA), lopinavir and ritonavir (donated by Abbott Laboratories, Illinois, IL, USA), Kaletra oral solution (80 mg/mL lopinavir and 20 mg/mL ritonavir; Abbott; obtained from dept. Pharmacy, Erasmus MC, Rotterdam, the Netherlands), nevirapine and tipranavir (donated by Boehringer Ingelheim, Ingelheim am Rhein, Germany), efavirenz (Moravek, Brea, CA, USA), metronidazole, metoprolol, piroxicam, amoxicillin, and carbamazepine (all from Sigma-Aldrich, Zwijndrecht, the Netherlands). α-cyano-4-hydroxycinnamic acid solution (Agilent Technologies; 6.2 mg/mL in methanol/acetonitrile/water 36/56/8 v/v/v, pH = 2.5). Sodium iodide (purity >99.99%, Sigma-Aldrich, Zwijndrecht, the Netherlands). Methanol (ULC/MS grade, Biosolve, Valkenswaard, the Netherlands). Phosphate-Buffered Saline (PBS; 1x; pH = 7.4; CaCl_2_ and MgCl_2_; product number 10010) was obtained from Invitrogen. Red blood cell lysis buffer was obtained from Roche Diagnostics (Mannheim, Germany, Cat. No. 11814389001).

### Stock solutions

All stock solutions for MALDI-triple quadrupole MS analysis were prepared in methanol by serial dilution. As internal standards we used: 20 µM nelfinavir (quantitation of lopinavir and ritonavir in plasma), 5 µM methotrexate (quantitation of saquinavir, nelfinavir, and indinavir in plasma), 2 µM nelfinavir (quantitation of lopinavir and ritonavir in 1×10^6^ PBMCs). To investigate the effect of co-medication on the quantitation performance, the following drug mixtures were added to the quality control samples: drug mixture A: 10 µM carbamazepine, metoprolol, metronidazole, amoxicillin, piroxicam, nevirapine, saquinavir, efavirenz, indinavir, and tipranavir (quantitation of lopinavir and ritonavir in plasma). Drug mixture B: 10 µM carbamazepine, metoprolol, metronidazole, amoxicillin, piroxicam, nevirapine, lopinavir, efavirenz, ritonavir, and tipranavir (quantitation of saquinavir, indinavir, and nelfinavir in plasma).

### Matrix preparation

For simultaneous quantitation of lopinavir and ritonavir, 10 µL 100 mM NaI in MeOH was added to 990 µL matrix solution (α-cyano-4-hydroxycinnamic acid). For simultaneous quantitation of saquinavir, indinavir, and nelfinavir, the matrix solution was diluted 1∶1 v/v in nanopure water.

### MALDI-triple quadrupole mass spectrometry

A FlashQuant Workstation equipped with a 4000 QTRAP mass analyzer (MDS Analytical Technologies, Concord, Ontario, Canada) was used for automated measurements of the samples. The spots were measured in the selected reaction monitoring mode (unit resolution) by moving the target plate in a straight horizontal line at a speed of 1 mm per second while firing of the laser at 1,000 Hz, which corresponds to an analysis time of 4.5 seconds per spot. For specific setting see Table 1. Analyst 1.4.2. and FlashQuant 1.0 software (MDS Analytical Technologies, Concord, Ontario, Canada) were used to operate the instrument and for automated data analysis. A calibration curve was constructed by plotting the analyte-to-internal standard area ratios against the concentrations spiked using a 1/concentration^2^ weighed linear curve. Quality controls were used to check the validity of the calibration curves. The absolute lower limit of quantification was set at the mean analyte area in the zero samples +10 times the standard deviation. Samples were spotted onto Opti-TOF 384 well stainless steel target plates (123×81 mm; MDS Analytical Technologies, Concord, Ontario, Canada).

**Table 1:** Instrument settings for quantitative analysis of HIV protease inhibitors.

compound	form	ion transition	CE (V)	CXP (V)	plate (V)	laser power (%)
lopinavir	sodiated	651.4 → 439.3	52	10	60	40
ritonavir	sodiated	743.3 → 573.3	50	10	60	40
nelfinavir	sodiated	590.4 → 338.2	50	10	60	40
saquinavir	protonated	671.4 → 570.4	44	8	65	35
indinavir	protonated	614.4 → 421.2	44	12	50	35
nelfinavir	protonated	568.4 → 330.2	45	8	50	35
methotrexate	protonated	455.2 → 308.2	30	8	50	35

The skimmer was set at 0 V, CAD gas at 6, source gas at 10. CE  =  collision energy. CXP  =  collision cell exit potential. Plate  =  target plate containing the samples. Dwell time for each ion transition was 10 ms.

### Plasma sample preparation for MALDI-triple quadrupole MS

Plasma samples were prepared in 1.5 mL Eppendorf safe-lock tubes: 10 µL plasma were added to methanol, mixed using a pipette, and spiked with internal standard, analytes, and additional drugs, depending on the type of sample. Calibrators: 70 µL MeOH +10 µL plasma +10 µL internal standard +10 µL analytes. Quality controls: 60 µL MeOH +10 µL plasma +10 µL internal standard +10 µL analytes +10 µL drug mixture. Unknowns: 80 µL MeOH +10 µL plasma +10 µL internal standard. Blank: 90 µL MeOH +10 µL plasma. Zero: 70 µL MeOH +10 µL plasma +10 µL internal standard +10 µL drug mixture. For quantitation of lopinavir and ritonavir, nelfinavir (20 µM solution) was used as internal standard. For quantitation of saquinavir, indinavir, and nelfinavir, methotrexate (5 µM solution) was used as internal standard. To the quality control samples for lopinavir and ritonavir quantitation, drug mixture “A” was spiked containing carbamazepine, metoprolol, metronidazole, amoxicillin, piroxicam, nevirapine, saquinavir, efavirenz, indinavir, and tipranavir (10 µM each). To the quality control samples for saquinavir, indinavir, and nelfinavir, drug mixture “B” was spiked containing carbamazepine, metoprolol, metronidazole, amoxicillin, piroxicam, nevirapine, lopinavir, efavirenz, ritonavir, and tipranavir (10 µM each). Samples were extracted for at least one hour at 5°C and were then centrifuged at 14,000 rpm at room temperature for 5 minutes. Subsequently, supernatants were mixed with matrix solution (1∶2, v/v; for matrix see Section “matrix preparation”), 0.75 µL matrix/sample mixture was deposited on three positions on the target plate and was dried at room temperature. MALDI-triple quadrupole MS measurements were carried out at the dept. Neurology (Erasmus MC, Rotterdam, the Netherlands).

### HPLC-UV

Lopinavir and ritonavir concentrations in plasma were determined using a validated HPLC-UV assay as described previously [24]. HPLC-UV measurements were performed at the dept. Clinical Pharmacy (UMC St. Radboud, Nijmegen, the Netherlands). For the cross-validation, the samples were prepared independently at the two sites, and analytes and internal standards were not exchanged between the laboratories.

### Patients and sampling

HIV-1 infected children included were from the outpatient clinic of the Erasmus MC – Sophia, Rotterdam, the Netherlands. Venous blood was drawn at a single time point during their routine 3-month visits at the outpatient clinic. For quantitation of lopinavir and ritonavir in plasma, blood was collected in lithium-heparinised tubes. Single time point plasma concentrations of lopinavir and ritonavir were determined during each visit at our outpatient clinic, which is part of the routine clinical care (HPLC-UV assay, dept Clinical Pharmacy UMC St. Radboud). Samples used for cross validation were obtained from an HIV-1 infected child who was admitted to our paediatric ward for determination of lopinavir and ritonavir plasma concentrations over a 12 hour time interval after observed intake of Kaletra. For quantitation of lopinavir and ritonavir in PBMCs, venous blood was collected in one or two Vacutainer CPT tubes (BD, NJ, USA). The Vacutainer CPT tubes were transported to the laboratory immediately after drawing of the blood sample.

For PBMCs treated with red blood cell lysis buffer (see below), PBMCs (and plasma) were collected from 16 HIV-1 infected children during one outpatient visit, and from one HIV-1 infected child PBMCs (and plasma) were collected during two consecutive visits at our outpatient clinic, i.e. a total of 17 PBMC samples were treated with red blood cell lysis buffer. Median age of the patients was 10.5 years old (25^th^ – 75^th^ percentile: 8.6 – 13.7). Eight patients were male, and nine were female. Fifteen patients received Kaletra + lamivudine + abacavir once daily and one patient received Kaletra + emtricitabine + tenofovir once daily. Samples were drawn at a median of 16 hours after Kaletra intake (25^th^ – 75^th^ percentile: 14.8 – 17.3). PBMC processing time (time between drawing of the blood sample and storage at -80°C) was 80 minutes (25^th^ – 75^th^ percentile: 75 – 90). For PBMCs not treated with red blood cell lysis buffer, PBMCs (and plasma) were collected from eight HIV-1 infected children during one outpatient clinic visit, i.e. eight PBMC samples were not treated with red blood cell lysis buffer. Median age of the patients was 10.5 years old (25^th^ – 75^th^ percentile: 8.9 – 13.0). Three patients were male, and five were female. Seven patients received Kaletra + lamivudine + abacavir once daily and one patient received Kaletra + emtricitabine + tenofovir once daily. Samples were drawn at a median of 14.5 hours after Kaletra intake (25^th^ – 75^th^ percentile: 13.3 – 16.5). PBMC processing time (time between drawing of the blood sample and storage at −80°C) was 78 minutes (25^th^ – 75^th^ percentile: 72 – 83).

### Isolation of PBMCs from HIV infected patients

The Vacutainer CPT tubes were processed immediately upon arrival in the laboratory. Tubes were centrifuged at 1750 g, brake 5, at room temperature for 15 minutes. The plasma layer containing the PBMCs was transferred to 15 mL polypropylene tubes (Falcon conical tubes; BD, NJ, USA) and ice-cold PBS was added until a volume of 12 mL was reached. Samples were centrifuged at 650 g with brake 9, at 4°C for 6 minutes. The supernatant was discarded and the cell pellet was loosened by gently ticking against the tube. Two mL of red blood cell lysis buffer (room temperature) was added, and cells were incubated for 1–2 minutes while gently swirling the cells. Ice-cold PBS was added until a volume of 12 mL was reached. For part of the samples, the red blood cell lysis buffer was not used. In that case, ice-cold PBS was directly added after loosening of the cell pellet. Samples were centrifuged at 350 g with brake 9, at 4°C for 6 minutes. The supernatant was discarded and ice-cold PBS was added until a volume of 12 mL was reached. Samples were centrifuged at 350 g, brake 9, at 4°C for 6 minutes. For part of the samples, 1 mL of supernatant was collected to investigate the amount of drugs present in the final wash step, and the remaining supernatants were discarded. We estimated that ∼20 µL washing fluid remained on top of the cell pellet after discarding the supernatant. Cells were then suspended in a small volume of ice-cold PBS, typically 200 – 400 µL depending on the estimated amount of cells isolated. Nucleated cells were counted by trypan blue dye exclusion and/or Türks using a Burker-Türk counting chamber and a light microscope. Cell viability was over 95%. Subsequently, ice-cold PBS was added until a concentration was reached of one million PBMC per 100 µL PBS. Cell suspensions were aliquoted as one million nucleated cells, i.e. PBMCs, in 100 µL per 1.5 mL polypropylene tube, and were stored at – 80°C.

### Isolation of PBMC from buffy coat

For preparation of the calibrators, quality controls, blanks, and zeros, PBMCs were isolated from a buffy coat (Sanquin, Rotterdam, the Netherlands) using a standard Ficoll density gradient. Nucleated cells were counted by trypan blue dye exclusion using a Burker-Türk cell chamber and a light microscope. Cell viability was over 95%. PBMCs were aliquoted in 1.5 mL polypropylene tubes at a concentration of 10 million cells in 500 µL PBS, and were subsequently stored at – 80°C.

### Preparation of PBMC from HIV infected patients

PBMC samples were thawed to room temperature. Subsequently, 300 µL MeOH was added and 20 µL internal standard (2 µM nelfinavir in MeOH). Samples were extracted for at least one hour at 5°C. Samples were then sonicated at 70% amplitude at −7°C for 10 seconds (Branson Digital Sonifier), and then further extracted at 5°C overnight. The next day, samples were centrifuged at 14,000 rpm at room temperature for 5 minutes, supernatants were collected and transferred to a 96-wells polypropylene plate. Nanopure water (800 µL) was added to each sample and the samples were processed using a 96-wells solid phase extraction (SPE) plate (Oasis HLB μelution plate, Waters Corporation, Milford, Massachusetts, USA). SPE wells were conditioned with 200 µL MeOH, and equilibrated with 200 µL nanopure water. Subsequently, the samples were loaded on to the SPE wells (600 µL twice). The wells were washed twice with 600 µL MeOH/nanopure water (1∶3, v/v), and the samples were eluted from SPE wells with 50 µL MeOH twice. The eluates were collected in a polypropylene microtiter plate, and dried using a SpeedVac (∼1 hour using a heated mantle at 45°C). Samples were then reconstituted in 10 µL matrix solution, 0.75 µL matrix/sample mixture was deposited on three positions on the target plate and dried at room temperature.

### Preparation of PBMCs obtained from buffy coat

PBMC samples (10 million PBMC in 500 µL PBS per vial) were thawed to room temperature, and 500 µL MeOH was added to each vial. Subsequently, samples were spiked with 20 µL internal standard (20 µM nelfinavir) and 20 µL analytes (Kaletra oral solution diluted in MeOH). Samples were then sonicated at 70% amplitude at −7°C for 10 seconds (Branson Digital Sonifier). The equivalent of one million PBMCs (104 µL) was transferred to a clean 1.5 mL polypropylene vial and 300 µL MeOH/PBS (5/1, v/v) was added. Samples were extracted at 5°C overnight and further processed as described above for the PBMC samples of HIV infected patients.

### Safety statement

Blood samples of HIV infected patients were transported in closed containers and were processed in a biosafety level 2 laboratory. Extraction of the plasma and PBMC samples in methanol for one hour inactivated infectious HIV as reported previously [24]–[26]. Further sample preparation steps and measurements were performed under less stringent safety conditions.

## Results

### Plasma

First we tested the MALDI-triple quadrupole MS approach on plasma samples, which is currently the standard type of sample for drug monitoring.

Protein precipitation of plasma with methanol provided sufficiently clean extracts for precise, accurate, and sensitive quantitative analysis of HIV protease inhibitors by MALDI-triple quadrupole MS (see below), and further preparation of the plasma samples with solid phase extraction was therefore not needed.

**Figure 1:**
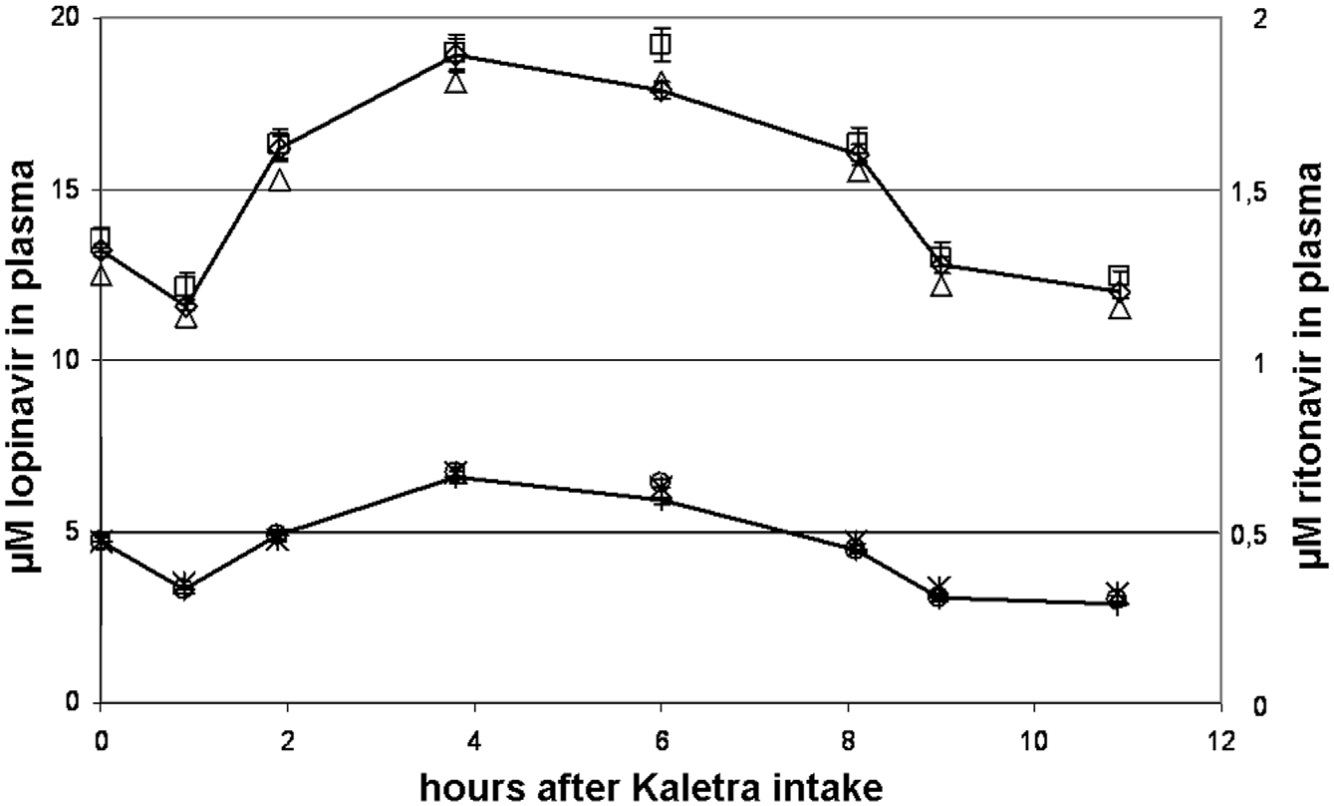
Lopinavir and ritonavir concentrations in an HIV infected child determined by HPLC-UV and by MALDI-triple quadrupole MS. Pharmacokinetic curve of lopinavir and ritonavir in one HIV infected child determined by HPLC-UV (triangles for lopinavir and crosses for ritonavir), MALDI-triple quadrupole MS (diamonds for lopinavir and circles for ritonavir), and MALDI-triple quadrupole MS when ten additional drugs (10 µM per drug) were spiked to the patient's samples (squares for lopinavir and pluses for ritonavir).

Simultaneous quantification of lopinavir and ritonavir was achieved using nelfinavir as internal standard, and SRMs were performed on the sodium adducts of these drugs. For these analyses, sodium iodide (NaI) was added to the matrix solution to increase the intensity of the sodium adducts of the drugs. For the simultaneous quantification of saquinavir, indinavir, and nelfinavir, we used methotrexate as internal standard and SRMs were performed on the protonated forms of the drugs (for instrument settings see Table 1). The lower limits of quantification (LLOQ) obtained in plasma were 167 nM for lopinavir, 14.5 nM for ritonavir, 16 nM for nelfinavir, 16 nM for indinavir, and 3.2 nM for saquinavir, and the precisions and accuracies were within the criteria set by the FDA for bioanalysis [27]. Co-medication did not affect the performance of the assays, as shown by spiking of the quality control samples with an additional ten drugs at a plasma concentration of 10 µM each (see Table S1). The MALDI-triple quadrupole MS assay was used to determine a pharmacokinetic curve of lopinavir and ritonavir in an HIV-1 infected child receiving Kaletra and two nucleoside reverse transcriptase inhibitors (NRTI) twice daily per os. Samples were also measured using a validated HPLC-UV assay (International Quality Control Program for Therapeutic Drug Monitoring in HIV infection, Department of Clinical Pharmacy, University Medical Center Nijmegen, the Netherlands) [24]. The two assays yielded similar results. For the MALDI-triple quadrupole MS assay, spiking of the patient's samples with an additional ten drugs (10 µM each) did not affect the results (see Figure 1 and Table S2).

### PBMCs

We developed a MALDI-triple quadrupole MS assay for simultaneous quantification of lopinavir and ritonavir in one million peripheral blood mononuclear cells (PBMC). The LLOQ was 834 nM for lopinavir and 73 nM for ritonavir, and the precisions and accuracies were within the criteria set by the FDA for bioanalysis (see Table S3). Storage of the sample on the target plate in a closed container at ambient conditions for up to one month analyzed did not affect the precisions, accuracies, or LLOQs (data not shown).

The intracellular concentrations were determined by taking into account that one averaged PBMC has an intracellular volume of 4×10^−13^ L. To assess the effect of solid phase extraction on the precisions and accuracies, we processed one set of plasma samples that were used for cross-validation with HPLC-UV using the solid phase extraction protocol for PBMCs, and we found that this step did not affect the precisions or the accuracies for lopinavir and ritonavir (data not shown). Shown in Table 2 are the precisions for biological replicates, i.e. analysis of multiple PBMC aliquots from HIV infected children (three aliquots per child).

**Table 2:** Repeat analysis of PBMC aliquots obtained from HIV-1 infected children.

		aliquot 1 (µM)			aliquot 2 (µM)			aliquot 3 (µM)			mean (µM)	%CV
patient	drug	spot 1	spot 2	spot 3	spot 1	spot 2	spot 3	spot 1	spot 2	spot 3		
1	LPV	2.61	2.70	2.70	2.71	2.84	2.73	2.80	3.14	2.90	2.79	5.6
2	LPV	4.66	5.34	5.01	3.97	4.22	4.01	4.48	4.00	4.88	4.51	11.1
3	LPV	2.11	2.05	2.08	2.43	2.48	2.47	2.13	2.24	2.65	2.29	9.5
1	RTV	0.299	0.279	0.322	0.290	0.278	0.277	0.340	0.313	0.309	0.301	7.3
2	RTV	0.495	0.478	0.469	0.401	0.427	0.396	0.427	0.443	0.459	0.444	7.7
3	RTV	0.336	0.351	0.346	0.336	0.347	0.359	0.375	0.354	0.369	0.352	3.8

For three HIV-1 infected children receiving Kaletra once daily, three aliquots of one million PBMC each were processed as described in the text (solid phase extraction followed by methanol extraction, and spotting in triplicate) to determine the overall precisions (%CV) of PBMC processing and sample measurements. Shown are the concentrations of lopinavir (LPV) and ritonavir (RTV) in µM as determined in a single spot on the target plate.

Assessment of the purity of the cells aliquoted showed that 4.6×10^5^ erythrocytes (sd 3.4×10^5^, n = 5) were present per aliquot of 1×10^6^ PBMC. Treatment of the cells isolated with red blood cell lysis buffer (2 minutes, room temperature) during the washing procedure resulted in >2 Log reduction in the number of erythrocytes per million PBMC (mean 3.8×10^3^ erythrocytes, sd 5.9×10^3^, n = 5). For eight HIV-1 infected children receiving Kaletra and 2 NRTIs once daily, we determined the concentrations of lopinavir and ritonavir in the final wash step and found that these constitute less than 4.5% (lopinavir) and less than 8.7% (ritonavir) of the amount measured in one aliquot of one million PBMC.

### Application

The MALDI-triple quadrupole MS assay was used to determine the intracellular concentrations of lopinavir and ritonavir in PBMC obtained from HIV-1 infected children receiving Kaletra (lopinavir + ritonavir) and two NRTIs once daily. Part of the samples was processed with red blood cell lysis buffer and part without to further investigate the effect of using this buffer on the concentrations determined. Plasma concentrations of lopinavir and ritonavir were determined by HPLC-UV, which is part of the routine clinical care in our setting. Blood samples were drawn at a single time point after medication intake. Plasma and intracellular concentrations of lopinavir and ritonavir are shown in Table 3, and the correlations are shown in Table 4.

**Table 3:** Plasma and intracellular concentrations of lopinavir and ritonavir in HIV-1 infected children.

	RBCLB+ (n = 17)	RBCLB- (n = 8)	p-value
µM lopinavir plasma	16.1 (9.8 – 21.2)	15.7 (10.3 – 19.7)	0.930
µM lopinavir PBMC	4.4 (2.8 – 6.0)	3.3 (3.0 – 7.1)	0.975
PBMC/plasma ratio lopinavir	0.34 (0.19 – 0.39)	0.26 (0.18 – 0.36)	0.634
µM ritonavir plasma	0.62 (0.36 – 1.17)	0.72 (0.42 – 0.90)	0.884
µM ritonavir PBMC	0.32 (0.28 – 0.35)	0.89 (0.49 – 1.23)	0.001
PBMC/plasma ratio ritonavir	0.46 (0.31 – 0.70)	1.53 (0.85 – 2.36)	0.004
Hours after intake	16.0 (14.8 – 17.3)	14.5 (13.3 – 16.5)	0.243
Age	10.5 (8.6 – 13.7)	10.5 (8.9 – 13.0)	0.861
PBMC processing time (minutes)	80 (75 – 90)	78 (72 – 83)	0.188

Values between brackets are expressed as medians (25^th^ percentile – 75^th^ percentile). P values were determined using a Mann Whitney U test. All patients administered Kaletra (lopinavir + ritonavir) +2 NRTIs once daily. RBCLB+ = PBMCs treated with red blood cell lysis buffer. RBCLB-  =  PBMCs not treated with red blood cell lysis buffer. The RBCLB+ group consisted of 16 HIV-1 infected children. For one child, concentrations were determined during two separate visits at the outpatient clinic. RBCLB- group consisted of 8 HIV-1 infected children. Three children were included in both the RBCLB+ group and RBCLB- group as for these patients samples were processed with and without RBCLB. Plasma concentrations were determined by HPLC-UV and intracellular concentrations by MALDI-triple quadrupole MS. For one patient in the RBCLB- group, intracellular concentration of lopinavir was below the LLOQ while the intracellular concentration of ritonavir was above the LLOQ. Hours after intake  =  time difference in hours between medication intake and drawing of the blood sample. PBMC processing time  =  time difference between drawing of the blood sample and aliquoting of the PBMC.

**Table 4:** Correlations between plasma and intracellular levels, and correlations between the concentrations of the two drugs in HIV-1 infected children.

	RBCLB+ (n = 17)	RBCLB- (n = 8)
lopinavir and ritonavir in plasma	0.973 (0.01)	0.952 (0.01)
lopinavir and ritonavir in PBMC	0.613 (0.01)	0.757 (0.05)
lopinavir in plasma and in PBMC	0.475 (NS)	0.613 (NS)
Ritonavir in plasma and in PBMC	0.510 (0.05)	0.500 (NS)
PBMC/plasma ratio lopinavir and PBMC/plasma ratio ritonavir	0.604 (0.05)	0.464 (NS)
lopinavir in plasma and in PBMC	0.531 (0.01) (RBCLB+ and RBCLB- combined)	
lopinavir and ritonavir in plasma	0.965 (0.01) (RBCLB+ and RBCLB- combined)	

Correlations were calculated using a Spearman correlation. Significance levels are reported between brackets. NS  =  not significant.

## Discussion

Liquid chromatography (LC) – based assays are widely used to determine drug concentrations in human samples, in particular assays that combine LC with UV-light detection or with detection by electrospray ionization (ESI) mass spectrometry (MS). Of these two platforms, LC-ESI-MS provides in general better sensitivities, a higher selectivity and shorter analysis times than LC-UV. Still, high throughput quantitative analysis is difficult to achieve using LC-based assays, because the most time-consuming part of the analysis, i.e. the LC-step, takes at least several minutes per sample. The LC-step is not necessarily needed for sensitive quantitative analysis of drugs in biological samples using MALDI mass spectrometry, because MALDI suffers less from ion suppression than ESI [23], [28]. The analysis time using MALDI-based assays is considerably shorter than using LC-based assays; in this case samples were measured at a speed of 13.5 seconds per sample (triplicate analysis), which is approximately 25 – 100 times faster than the LC-based approaches [2], [29]–[31]. The LLOQs in PBMCs for our MALDI-MS assay were 25 femtomole per spot for lopinavir and 2.2 femtomole per spot for ritonavir, which is comparable to the LLOQs in PBMCs of 6 femtomole on column for lopinavir and 5 femtomole on column for ritonavir for the LC-ESI-MS assay described by Colombo et al. [2]. Runtime of that assay was 20 minutes, and accuracies were still below the recommended 15% when a variable amount of input material, i.e. between 1.4×10^6^ PBMCs and 9.6×10^6^ PBMCs, was used for the assay. Pèlerin et al. described an LC-ESI-MS assay with an LLOQ of 70 femtomole per µL intracellular volume for ritonavir and 80 femtomole per µL intracellular volume for lopinavir [30]. The amount of PBMCs isolated from 7 mL blood was used as input material for the assay. Runtime of the assay was 20 minutes. Ter Heine et al. described an LC-ESI-MS assay with LLOQ of 1.9 femtomole/3×10^6^ PBMCs for lopinavir and an LLOQ of 1.7 femtomole/3×10^6^ PBMCs for ritonavir [31]. Runtime of the assay was 10 minutes. For clinical samples, an aliquot of 3×10^6^ PBMCs was extracted in a volume of 200 µL, and 30 µL extract was used for LC-ESI-MS analysis. Ehrardt et al. described an LC-ESI-MS method with an LLOQ of 139 fmol/3×10^6^ PBMCs for ritonavir and 159 fmol/3×10^6^ PBMCs for lopinavir [29]. Runtime of the assay was 6 minutes. After sample preparation of 3×10^6^ PBMCs, the dried extracts were reconstituted in 200 µL LC mobile phase and 40 µL were used for LC-ESI-MS measurements. LC-ESI-MS methods published thusfar have not been validated specifically for quantification of lopinavir and ritonavir in 1×10^6^ PBMCs. Yet, the sensitivities of the assays described in literature are likely to be sufficient for that purpose. In this study, we specifically validated our assay for quantification of lopinavir and ritonavir in 1×10^6^ PBMCs, because of our interest in pharmacokinetics of antitretroviral drugs in children. For the same reason, we specifically validated our assay for quantification of HIV protease inhibitors in 10 µL plasma.

We used the Selected Reaction Monitoring (SRM) mode of the MALDI – triple quadrupole mass spectrometer to circumvent background signals derived from the biological sample and from the matrix. In the SRM-mode of a triple quadrupole mass analyzer, only ions with masses corresponding to the mass of the compound of interest are able to pass the first quadrupole, in the second quadrupole the ions collide with a gas resulting in fragmentation, and in the third quadrupole only the fragment ions are able to pass whose masses correspond with the mass of a specific fragment of the drug of interest. The fragmentation pattern of an ion is highly structure specific, and isobaric ions derived from the matrix or from the biological sample are not able to pass through the triple quadrupole mass analyzer and are thus not detected. In addition, the SRM-mode enhances the sensitivity of the measurements and extends the linear dynamic range [17]. We have previously shown that MALDI-triple quadrupole is a suitable technique to quantify HIV protease inhibitors in PBMC extracts [20]. In this study, we show clinical application as well as quantitative analysis of HIV protease inhibitors in plasma including a cross-validation, and we used a different matrix, i.e. α-cyano-4-hydroxycinnamic acid, which resulted in better precisions and higher sensitivities. Furthermore, we show that the MALDI-triple quadrupole MS approach allows for multi-target quantification using a chemical analogue as internal standard, and that samples co-crystallized with matrix are stable at ambient conditions for up to a month. Thus, samples can be stored and measured multiple times with the same or a different type of MALDI mass spectrometer. In this study, we used HIV protease inhibitors as model compounds, but the approach is also applicable to other drugs. For example, we were able to quantify methotrexate down to 5 nM in plasma (data not shown).

The sample preparation techniques used prior to MALDI-triple quadrupole MS analysis allow for fast and automated processing of large numbers of samples, e.g. in 96-wells plate format [23]. Normally, these sample preparation techniques are also used for subsequent analysis by LC-based assays. Plasma and cells were extracted in methanol, which simultaneously inactivates infectious HIV [25], [26]. For plasma, no further sample preparation steps were needed, i.e. the lysates were mixed with matrix solution and deposited on a target plate. Drying of the plasma lysates after protein precipitation and subsequent reconstitution in a small volume of matrix solution resulted in poor crystallization of the samples and severe loss in sensitivity, which is in agreement with two previous reports [16], [22]. In our approach, the plasma samples are simply diluted 30 times, which results in good crystallization and highly sensitive measurements. For PBMC samples, we found that this methanol dilution step did not result in the sensitivity needed for intracellular quantitative analysis of HIV protease inhibitors in clinical samples, and therefore samples were further processed with solid phase extraction.

The MALDI-triple quadrupole MS assay was used to investigate the intracellular concentrations of lopinavir and ritonavir in relation to their plasma concentrations in HIV-1 infected children receiving Kaletra, a co-formulation of the HIV protease inhibitors lopinavir and ritonavir (4∶1, w/w), once daily. Both drugs are metabolized in the liver by CYP3A4 isoenzymes. Ritonavir inhibits the CYP3A4-mediated metabolism, thereby enhancing the pharmacokinetic properties of lopinavir [32]. This is in agreement with the strong correlation (ρ = 0.97, p = 0.01) that we found between the plasma concentrations of lopinavir and the plasma concentrations of ritonavir in HIV-1 infected children. Ribera et al. also found a correlation between the plasma AUC of lopinavir and ritonavir in HIV-1 infected adults, although this correlation was less pronounced (r = 0.63, p<0.001) [33].

Whether lopinavir and ritonavir enter the cellular compartment in blood by passive diffusion, active transport, or a combination of both, is currently unknown. Passive diffusion is likely to occur as lopinavir and ritonavir are both lipophilic drugs with an octanol/saline partition coefficient of 214 and 22.2, respectively, and are thus able to cross the plasma membrane and enter the cells. Active transport into cells has not been reported yet. On the other hand, lopinavir and ritonavir are substrates for drug efflux pumps, although no correlation has been found between drug efflux pumps and intracellular concentrations of HIV protease inhibitors in HIV infected patients [5], [10]. It should be noted that only unbound non-ionized drugs can cross the cell membrane by passive diffusion. In vivo, the unbound concentrations of lopinavir and ritonavir are only 1–2% of the total amount in plasma. In vitro studies have already shown that the high protein binding significantly affects the accumulation of protease inhibitors [34], [35]. It is expected that lopinavir and ritonavir are also highly bound to proteins within cells. To which proteins and to what extent is currently unknown.

Disposition studies of lopinavir and ritonavir in rats showed that both HIV protease inhibitors penetrate poorly into the cellular fraction of blood. Literature indicates a whole blood/plasma ratio of 0.5 for lopinavir and a whole blood/plasma ratio of 0.26 to 0.68 for ritonavir [36], [37]. Koal et al. also reported lower concentrations of HIV protease inhibitors in whole blood compared to plasma in HIV infected patients [38]. This shows that lopinavir and ritonavir penetrate poorly into the cellular fraction of blood. It should be noted that the cellular fraction of whole blood consists for >99% of erythrocytes, and that differences may exist between penetration in erythrocytes and in PBMC.

Accumulation of lopinavir [1], [4] and ritonavir [4] in PBMC has been reported in HIV-1 infected adults receiving Kaletra BID: Breilh et al. reported a PBMC/plasma ratio of lopinavir of 3.2 (month 1) and 2.4 (month 6) in patients who responded to therapy, and 2.3 (month 1) and 1.4 (month 6) in patients who failed on therapy [1]. Ritonavir concentrations were not determined in this study. Crommentuyn et al. reported a PBMC/plasma ratio of 1.18 for lopinavir and 4.59 for ritonavir in HIV-1 infected adults receiving Kaletra BID [4]. Ritonavir accumulation was also reported by Khoo et al. They observed a PBMC/plasma ratio of 1 when ritonavir is administered as sole protease inhibitor, 1.25 using ritonavir-boosted saquinavir, and 1.8 using ritonavir-boosted indinavir [39]. On the other hand, Colombo et al. reported a PBMC/plasma ratio of 0.55 for lopinavir in HIV infected adults [2], [3]. Ehrhardt et al. showed in one healthy volunteer that, after a single dose of Kaletra, lopinavir concentrations were lower in PBMC than in plasma, but ritonavir concentrations were higher in PBMC than in plasma [29].

In this study, we found lower lopinavir concentrations in PBMC than in plasma in HIV-1 infected children receiving Kaletra once daily. For ritonavir, accumulation in PBMC was highly dependent on the sample preparation used. When erythrocytes were removed by RBCLB no accumulation was found, while accumulation was found when not using RBCLB. This shows that sample preparation can have a major influence on the concentrations measured. This step was included in the sample preparation protocol because of the erythrocytes present in cell suspensions after Ficoll density gradient centrifugation and subsequent thorough washing of the cells. Although cells were incubated for only two minutes in this buffer, we cannot exclude efflux of ritonavir because this incubation occurred at room temperature. However, this does not explain the lower PBMC concentrations compared to plasma for lopinavir as there were no significant differences in the PBMC/plasma ratio when PBMC were prepared with RBCLB or without RBCLB.

In our opinion, the higher PBMC/plasma ratios when RBCLB is not used is not due to high intracellular concentrations of ritonavir in erythrocytes. Previous studies have already shown poor penetration of this drug in blood cells (see above). In addition, when RBCLB was not used, the erythrocyte/PBMC ratio in the cell preparation was ∼1∶2 (count/count), and the intracellular volume of an erythrocyte is lower (∼80 fL) compared to that of an average PBMC (∼400 fL). An explanation might be that, due to erythrocyte lysis, proteins are released into the lysis buffer and that ritonavir bound to proteins on the outside of the plasma membrane now bind to the erythrocyte-derived proteins in the buffer. On the other hand, this effect was not observed for lopinavir. Ritonavir but also lopinavir are highly bound in plasma to albumin and α1-acid glycoprotein [32]. In vitro studies have already shown that higher protein concentrations in cell cultures result in significantly lower intracellular concentrations [34], [35].

We found a correlation between the intracellular concentrations of lopinavir and the intracellular concentrations of ritonavir (ρ = 0.61, p = 0.01 and ρ = 0.76, p = 0.05; RBCLB+ and RBCLB-, respectively). This is in agreement with previous reports on ritonavir-boosted saquinavir and ritonavir-boosted indinavir [5], [39], [40]. To our best knowledge this is the first time such correlation is shown for Kaletra. We found that the correlation between the plasma concentrations and intracellular concentrations of lopinavir (ρ = 0.53, p = 0.01) or ritonavir (ρ = 0.51, p = 0.05 and ρ = 0.5, NS; RBCLB+ and RBCLB- respectively) were less pronounced. Breilh et al. showed for HIV-1 infected adults receiving Kaletra BID that the plasma concentrations of lopinavir correlated with the intracellular concentrations of lopinavir after one month of treatment (r^2^ = 0.72, p<10^−6^). Yet, this correlation was not found after six months of treatment (r^2^ = 0.17, p = 0.3) [1]. For ritonavir, this correlation was not assessed. For ritonavir-boosted saquinavir, the plasma concentrations of ritonavir correlated poorly to the intracellular concentrations: r^2^ = 0.33; p = 0.053 and r^2^ = 0.31, p = 0.06) [5], [40]. Above shows that lopinavir levels and ritonavir levels correlate to each other within the same compartment, i.e. plasma or PBMC, but also that for each drug the correlation between these compartments is poor.

In conclusion, we have developed a novel assay for the fast and sensitive quantification of compounds that can be applied to monitor drugs in plasma and intracellularly in treatment of various illnesses including antiviral treatment.

## Supporting Information

Table S1 Quantitative analysis of HIV protease inhibitors in plasma. The first column shows the specific drug and the second column shows the concentration of the drug spiked in plasma in µM. The third column shows the actual amount of drug in femtomoles (fmol) in a single spot on the target plate. The fourth column shows the accuracies, expressed as % deviation, for samples used to construct the calibration curve (calibrators), and the fifth column show the accuracies for samples used to test the validity of the calibration curve (quality controls). Precisions, expressed as %CV, are reported between brackets (n = 9 spots on the target plate). For the analysis of lopinavir and ritonavir, quality controls were spiked with carbamazepine, metoprolol, metronidazol, amoxicillin, piroxicam, nevirapine, saquinavir, efavirenz, indinavir, and tipranavir at a plasma concentration of 10 µM each. For analysis of nelfinavir, saquinavir, and indinavir, quality controls were spiked with efavirenz, tipranavir, lopinavir, ritonavir, carbamazepine, metoprolol, metronidazol, amoxicillin, prioxicam, and nevirapine at a plasma concentration of 10 µM each. These drugs were not added to the calibrators. The calibrators were prepared in plasma from a different healthy donor than the quality controls were.
(0.06 MB DOC)

Table S2 Lopinavir and ritonavir concentrations in plasma in an HIV-1 infected child. The first column shows the time points in hours at which blood samples were collected from one HIV-1 infected child after observed intake of Kaletra. The second column shows the components of Kaletra, i.e. lopinavir and ritonavir. The third column shows the concentration of the compounds in µM determined by HPLC-UV. The fourth and fifth columns show the concentration of the compounds in µM determined by MALDI-triple quadrupole MS. Three technical replicates were measured for each sample, i.e. three spots on the target plate. The precisions (%CV) are reported between brackets. The sixth and seventh columns show the deviations in percentage between the concentration of the compounds determined by MALDI-triple quadrupole MS and by HPLC-UV. For the MALDI-triple quadrupole MS analyses, one set of samples was spiked with an additional ten drugs (“drugs added”) and one set was not (“no drugs added”). The additional ten drugs spiked were carbamazepine, metoprolol, metronidazol, amoxicillin, piroxicam, nevirapine, saquinavir, efavirenz, indinavir, and tipranavir at a plasma concentration of 10 µM each.
(0.05 MB DOC)

Table S3 Quantitative analysis of lopinavir and ritonavir in one million PBMC. The regression coefficient were >0.998.
(0.04 MB DOC)
