# Correction: Ultra-Fast Analysis of Plasma and Intracellular Levels of HIV Protease Inhibitors in Children: A Clinical Application of MALDI Mass Spectrometry

**DOI:** 10.1371/annotation/9e6e8b98-cc08-47be-9ab1-3f1928e12fed

**Published:** 2010-07-23

**Authors:** Jeroen J. A. van Kampen, Mariska L. Reedijk, Peter C. Burgers, Lennard J. M. Dekker, Nico G. Hartwig, Ineke E. van der Ende, Ronald de Groot, Albert D. M. E. Osterhaus, David M. Burger, Theo M. Luider, Rob A. Gruters

There was an error in affiliation 6 for author David M. Burger. Affiliation 6 should be: Department of Pharmacy, Radboud University Nijmegen Medical Center, Nijmegen, The Netherlands. 

