# Supplementary material for: Ultra-Fast Analysis of Plasma and Intracellular Levels of HIV Protease Inhibitors in Children: A Clinical Application of MALDI Mass Spectrometry
Source: PLoS One. 2010 Jul 1;5(7):e11409. doi: 10.1371/journal.pone.0011409 (PMC2895665; doi:10.1371/journal.pone.0011409)
Supplement: Table S1 — Quantitative analysis of HIV protease inhibitors in plasma. The first column shows the specific drug and the second column shows the concentration of the drug spiked in plasma in µM. The third column shows the actual amount of drug in femtomoles (fmol) in a single spot on the target plate. The fourth column shows the accuracies, expressed as % deviation, for samples used to construct the calibration curve (calibrators), and the fifth column show the accuracies for samples used to test the validity of the calibration curve (quality controls). Precisions, expressed as %CV, are reported between brackets (n = 9 spots on the target plate). For the analysis of lopinavir and ritonavir, quality controls were spiked with carbamazepine, metoprolol, metronidazol, amoxicillin, piroxicam, nevirapine, saquinavir, efavirenz, indinavir, and tipranavir at a plasma concentration of 10 µM each. For analysis of nelfinavir, saquinavir, and indinavir, quality controls were spiked with efavirenz, tipranavir, lopinavir, ritonavir, carbamazepine, metoprolol, metronidazol, amoxicillin, prioxicam, and nevirapine at a plasma concentration of 10 µM each. These drugs were not added to the calibrators. The calibrators were prepared in plasma from a different healthy donor than the quality controls were. (0.06 MB DOC) [file pone.0011409.s001.doc]

Table S1. Quantitative analysis of HIV protease inhibitors in plasma.

|  |  |  | **calibrators** | **quality controls** |
| --- | --- | --- | --- | --- |
| **compound** | **μM in plasma** | **fmol per spot** | **% deviation (%CV)** | **% deviation (%CV)** |
| lopinavir | 40.71 | 1017.8 | 1.1 (1.3) | 4.6 (1.0) |
| lopinavir | 16.28 | 407 | 1.2 (2.3) | -1.1 (2.2) |
| lopinavir | 6.51 | 162.8 | -1.7 (2.8) | -0.5 (1.8) |
| lopinavir | 2.61 | 65.3 | -2.2 (1.9) | 0.0 (2.7) |
| lopinavir | 1.04 | 26 | 2.0 (5.2) | 3.9 (3.4) |
| lopinavir | 0.417 | 10.4 | -0.4 (9.2) | 6.0 (6.9) |
| lopinavir | 0.167 | 4.2 | 0.0 (9.0) | -4.3 (8.6) |
|  |  |  |  |  |
| ritonavir | 8.88 | 222 | -0.2 (2.2) | 5.7 (1.3) |
| ritonavir | 3.55 | 88.8 | 2.0 (2.1) | 0.2 (1.6) |
| ritonavir | 1.42 | 35.5 | -0.6 (1.7) | 0.5 (1.5) |
| ritonavir | 0.568 | 14.2 | -1.3 (2.4) | 1.3 (3.0) |
| ritonavir | 0.227 | 5.7 | -0.3 (3.4) | 5.9 (4.5) |
| ritonavir | 0.0909 | 2.3 | 1.6 (2.5) | 4.6 (3.1) |
| ritonavir | 0.0364 | 0.91 | -1.7 (8.1) | 5.2 (8.0) |
| ritonavir | 0.0145 | 0.36 | 0.5 (12.2) | 7.3 (11.0) |
|  |  |  |  |  |
| nelfinavir | 10 | 250 | -6.8 (4.3) | -13.3 (2.7) |
| nelfinavir | 2 | 50 | -4.4 (2.5) | -7.9 (2.6) |
| nelfinavir | 0.4 | 10 | 4.8 (4.1) | -4.7 (2.4) |
| nelfinavir | 0.08 | 2 | 8.1 (4.2) | -3.1 (4.1) |
| nelfinavir | 0.016 | 0.4 | -1.8 (4.4) | -10.1 (11.0) |
|  |  |  |  |  |
| indinavir | 10 | 250 | -4.4 (3.7) | -6.9 (2.4) |
| indinavir | 2 | 50 | -2.2 (1.8) | -5.0 (2.6) |
| indinavir | 0.4 | 10 | 2.6 (3.6) | -2.4 (3.8) |
| indinavir | 0.08 | 2 | 5.2 (4.7) | 2.1 (4.2) |
| indinavir | 0.016 | 0.4 | -1.1 (7.4) | -1.9 (12.0) |
|  |  |  |  |  |
| saquinavir | 10 | 250 | -5.2 (5.1) | -9.9 (3.9) |
| saquinavir | 2 | 50 | -5.3 (2.1) | -8.8 (2.8) |
| saquinavir | 0.4 | 10 | 1.2 (2.8) | -6.3 (2.4) |
| saquinavir | 0.08 | 2 | 5.2 (3.7) | -6.9 (3.3) |
| saquinavir | 0.016 | 0.4 | 5.5 (7.4) | 2.4 (6.2) |
| saquinavir | 0.0032 | 0.08 | -1.3 (10.5) | -8.8 (14.9) |

The first column shows the specific drug and the second column shows the concentration of the drug spiked in plasma in μM. The third column shows the actual amount of drug in femtomoles (fmol) in a single spot on the target plate. The fourth column shows the accuracies, expressed as % deviation, for samples used to construct the calibration curve (calibrators), and the fifth column show the accuracies for samples used to test the validity of the calibration curve (quality controls). Precisions, expressed as %CV, are reported between brackets (n= 9 spots on the target plate). For the analysis of lopinavir and ritonavir, quality controls were spiked with carbamazepine, metoprolol, metronidazol, amoxicillin, piroxicam, nevirapine, saquinavir, efavirenz, indinavir, and tipranavir at a plasma concentration of 10 μM each. For analysis of nelfinavir, saquinavir, and indinavir, quality controls were spiked with efavirenz, tipranavir, lopinavir, ritonavir, carbamazepine, metoprolol, metronidazol, amoxicillin, prioxicam, and nevirapine at a plasma concentration of 10 μM each. These drugs were not added to the calibrators. The calibrators were prepared in plasma from a different healthy donor than the quality controls were.
