# Supplementary material for: Ultra-Fast Analysis of Plasma and Intracellular Levels of HIV Protease Inhibitors in Children: A Clinical Application of MALDI Mass Spectrometry
Source: PLoS One. 2010 Jul 1;5(7):e11409. doi: 10.1371/journal.pone.0011409 (PMC2895665; doi:10.1371/journal.pone.0011409)
Supplement: Table S2 — Lopinavir and ritonavir concentrations in plasma in an HIV-1 infected child. The first column shows the time points in hours at which blood samples were collected from one HIV-1 infected child after observed intake of Kaletra. The second column shows the components of Kaletra, i.e. lopinavir and ritonavir. The third column shows the concentration of the compounds in µM determined by HPLC-UV. The fourth and fifth columns show the concentration of the compounds in µM determined by MALDI-triple quadrupole MS. Three technical replicates were measured for each sample, i.e. three spots on the target plate. The precisions (%CV) are reported between brackets. The sixth and seventh columns show the deviations in percentage between the concentration of the compounds determined by MALDI-triple quadrupole MS and by HPLC-UV. For the MALDI-triple quadrupole MS analyses, one set of samples was spiked with an additional ten drugs (“drugs added”) and one set was not (“no drugs added”). The additional ten drugs spiked were carbamazepine, metoprolol, metronidazol, amoxicillin, piroxicam, nevirapine, saquinavir, efavirenz, indinavir, and tipranavir at a plasma concentration of 10 µM each. (0.05 MB DOC) [file pone.0011409.s002.doc]

Table S2. Lopinavir and ritonavir concentrations in plasma in an HIV-1 infected child.

|  |  |  | **no drugs added** | **drugs added** | **no drugs added** | **drugs added** |
| --- | --- | --- | --- | --- | --- | --- |
| **time (h)** | **compound** | **HPLC-UV** | **MALDI (%CV)** | **MALDI (%CV)** | **% deviation** | **% deviation** |
| 0 | lopinavir | 12.5 | 13.2 (1.5) | 13.5 (2.7) | 5.7 | 8.4 |
|  | ritonavir | 0.47 | 0.47 (3.2) | 0.47 (4.1) | -1.1 | 0.2 |
| 1 | lopinavir | 11.3 | 11.6 (1.4) | 12.1 (3.8) | 3.0 | 6.8 |
|  | ritonavir | 0.35 | 0.33 (2.2) | 0.33 (3.6) | -6.6 | -5.1 |
| 2 | lopinavir | 15.3 | 16.2 (2.2) | 16.3 (2.7) | 5.8 | 6.8 |
|  | ritonavir | 0.48 | 0.49 (3.7) | 0.49 (2.1) | 1.6 | 2.6 |
| 4 | lopinavir | 18.1 | 18.9 (2.6) | 19.0 (2.7) | 4.3 | 5.2 |
|  | ritonavir | 0.67 | 0.66 (2.9) | 0.67 (2.9) | -1.0 | -0.4 |
| 6 | lopinavir | 18.1 | 17.9 (1.4) | 19.2 (2.5) | -1.2 | 6.2 |
|  | ritonavir | 0.63 | 0.59 (1.7) | 0.64 (1.8) | -6.6 | 1.4 |
| 8 | lopinavir | 15.5 | 16.0 (1.8) | 16.3 (3.1) | 3.3 | 5.0 |
|  | ritonavir | 0.47 | 0.45 (2.3) | 0.45 (3.2) | -3.4 | -4.1 |
| 9 | lopinavir | 12.2 | 12.8 (2.0) | 13.0 (3.3) | 5.2 | 6.8 |
|  | ritonavir | 0.34 | 0.31 (2.0) | 0.31 (4.0) | -7.6 | -7.5 |
| 11 | lopinavir | 11.5 | 12.0 (1.7) | 12.4 (1.8) | 4.2 | 8.2 |
|  | ritonavir | 0.32 | 0.29 (2.5) | 0.30 (5.4) | -10.1 | -5.8 |

The first column shows the time points in hours at which blood samples were collected from one HIV-1 infected child after observed intake of Kaletra. The second column shows the components of Kaletra, i.e. lopinavir and ritonavir. The third column shows the concentration of the compounds in μM determined by HPLC-UV. The fourth and fifth columns show the concentration of the compounds in μM determined by MALDI-triple quadrupole MS. Three technical replicates were measured for each sample, i.e. three spots on the target plate. The precisions (%CV) are reported between brackets. The sixth and seventh columns show the deviations in percentage between the concentration of the compounds determined by MALDI-triple quadrupole MS and by HPLC-UV. For the MALDI-triple quadrupole MS analyses, one set of samples was spiked with an additional ten drugs (“drugs added”) and one set was not (“no drugs added”). The additional ten drugs spiked were carbamazepine, metoprolol, metronidazol, amoxicillin, piroxicam, nevirapine, saquinavir, efavirenz, indinavir, and tipranavir at a plasma concentration of 10 μM each.
