# Supplementary material for: Ultra-Fast Analysis of Plasma and Intracellular Levels of HIV Protease Inhibitors in Children: A Clinical Application of MALDI Mass Spectrometry
Source: PLoS One. 2010 Jul 1;5(7):e11409. doi: 10.1371/journal.pone.0011409 (PMC2895665; doi:10.1371/journal.pone.0011409)
Supplement: Table S3 — Quantitative analysis of lopinavir and ritonavir in one million PBMC. The regression coefficient were >0.998. (0.04 MB DOC) [file pone.0011409.s003.doc]

Table S3. Quantitative analysis of lopinavir and ritonavir in one million PBMC.

|  |  |  |  | **calibrators (n=3)** | **quality controls (n=33)** |
| --- | --- | --- | --- | --- | --- |
| **compound** | **μM in PBMC** | **pmol 1x10^6^ PBMC** | **fmol per spot** | **% deviation (%CV)** | **% deviation (%CV)** |
| lopinavir | 203.5 | 81.4 | 6105 | - 4.7 (4.0) | - 7.3 (7.2) |
| lopinavir | 81.4 | 32.6 | 2442 | - 1.1 (0.9) | - 0.9 (7.3) |
| lopinavir | 32.6 | 13.0 | 977 | - 1.0 (1.2) | 2.1 (8.1) |
| lopinavir | 13.0 | 5.2 | 391 | 5.4 (2.7) | 2.4 (8.1) |
| lopinavir | 5.2 | 2.1 | 156 | 0.8 (2.4) | 1.2 (6.9) |
| lopinavir | 2.1 | 0.834 | 63 | 1.6 (2.4) | - 2.4 (11.5) |
| lopinavir | 0.834 | 0.334 | 25 | - 1.1 (9.6) | - 2.5 (17.5) |
|  |  |  |  |  |  |
| ritonavir | 44.4 | 17.8 | 1335 | - 6.6 (4.2) | - 8.4 (6.8) |
| ritonavir | 17.8 | 7.1 | 534 | - 1.1 (0.2) | - 2.3 (5.6) |
| ritonavir | 7.1 | 2.8 | 214 | - 0.8 (1.4) | 1.1 (5.8) |
| ritonavir | 2.8 | 1.1 | 85 | 2.9 (1.7) | 2.0 (4.9) |
| ritonavir | 1.1 | 0.455 | 34 | 0.3 (1.2) | 1.1 (5.3) |
| ritonavir | 0.455 | 0.182 | 14 | 5.2 (2.7) | 0.6 (7.4) |
| ritonavir | 0.182 | 0.073 | 5.5 | 1.5 (2.2) | - 2.1 (7.2) |
| ritonavir | 0.073 | 0.029 | 2.2 | - 1.5 (6.2) | - 13.4 (13.7) |

The regression coefficient were > 0.998
